# Early Neonatal Death in Harlequin Ichthyosis: A Case Report and Literature Review

**DOI:** 10.1002/ccr3.71190

**Published:** 2025-10-15

**Authors:** Ahmed Alanzi, Dawood Alatefi, Malik Alkabazi, Bano Alsaleh, Amir Fouad, Islam Al Oraby

**Affiliations:** ^1^ Anaesthesia and Pain Management Department King Hamad University Hospital Muharraq Bahrain; ^2^ Faculty of Medicine, University of Jordan Amman Jordan; ^3^ School of Dentistry, Khalij Libya Tripoli Libya; ^4^ Radiology Department King Hamad University Hospital Muharraq Bahrain; ^5^ Obstetrics and Gynecology Department Maternity & Children's Hospital Najran Kingdom of Saudi Arabia

**Keywords:** collodion baby, congenital ichthyosis, genetic counseling, Harlequin ichthyosis, neonatal intensive care, retinoid therapy

## Abstract

Harlequin ichthyosis is a rare, life‐threatening neonatal disorder often mistaken for collodion baby. We report a 37‐week neonate with severe ectropion, eclabium, and thick fissured scales who died on Day 2 despite optimal care. This case highlights the diagnostic challenges, intensive management needs, and poor prognosis of Harlequin ichthyosis.

**Summary**

- Harlequin ichthyosis is the most severe form of congenital ichthyosis, often initially mistaken for a collodion baby.
- Early recognition is critical to initiate intensive neonatal care, including humidity control, emollients, and infection prevention.
- Awareness of oral acitretin therapy and timely parental counseling can improve outcomes and guide future pregnancies.

## 1 Introduction

Harlequin ichthyosis (HI) is a rare, life‐threatening autosomal recessive keratinization disorder and represents the most severe form of congenital ichthyosis [1, 2]. It was first described by Oliver Hart in 1750 and historically carried an almost universally lethal prognosis, with most affected neonates dying within the first days of life due to skin barrier failure and overwhelming sepsis [3]. Clinically, HI is characterized by a striking armor‐like encasement of thick, yellowish hyperkeratotic plates separated by deep erythematous fissures, which distort the face and extremities [2]. Typical craniofacial features include severe ectropion, eclabium, a flattened nose, and poorly formed ears, often accompanied by limb pseudocontractures caused by the rigid skin [1].

The disease is caused by biallelic pathogenic variants in the *ABCA12* gene on chromosome 2q35, which encodes an ATP‐binding cassette (ABC) lipid transporter essential for normal lamellar granule function and stratum corneum lipid barrier formation [4]. Loss‐of‐function mutations, usually truncating alleles, result in defective epidermal lipid transport, failure of the intercellular lipid layers to form, and the characteristic thickened, fissured skin [5]. In contrast, missense variants in *ABCA12* typically lead to milder forms of autosomal recessive congenital ichthyoses, such as lamellar ichthyosis and congenital ichthyosiform erythroderma [1].

Despite advances in neonatal intensive care and the introduction of systemic retinoids, HI still carries a guarded prognosis, particularly in low‐resource settings [3]. Herein, we report a case of neonatal HI initially suspected as a collodion baby, who died on the second day of life despite intensive supportive measures.

## 2 Case Presentation

### 2.1 History and Physical Examination

A 38‐year‐old female, gravida 6 para 4, with a singleton intrauterine pregnancy at 27 weeks and 4 days of gestation and an estimated fetal weight of 1025 g presented to the obstetrics department for a routine antenatal visit. She had no complaints, and fetal movements were normal. Her obstetric history included four previous normal vaginal deliveries, with the last delivery 7 years earlier while she was on combined oral contraceptives. She had no significant medical or surgical history, was a nonsmoker, nonalcoholic, and was not on any chronic medications.

On examination, her vital signs were normal, and abdominal examination revealed a gravid uterus compatible with gestational age. Initial obstetric ultrasound demonstrated a breech presentation, oligohydramnios, and a posterior placenta. No fetal structural anomalies were noted, and the fetal face could not be visualized due to fetal position. She was scheduled for weekly Doppler ultrasound surveillance and prescribed low‐molecular‐weight heparin (Clexane 4000 IU daily).

**FIGURE 1:**
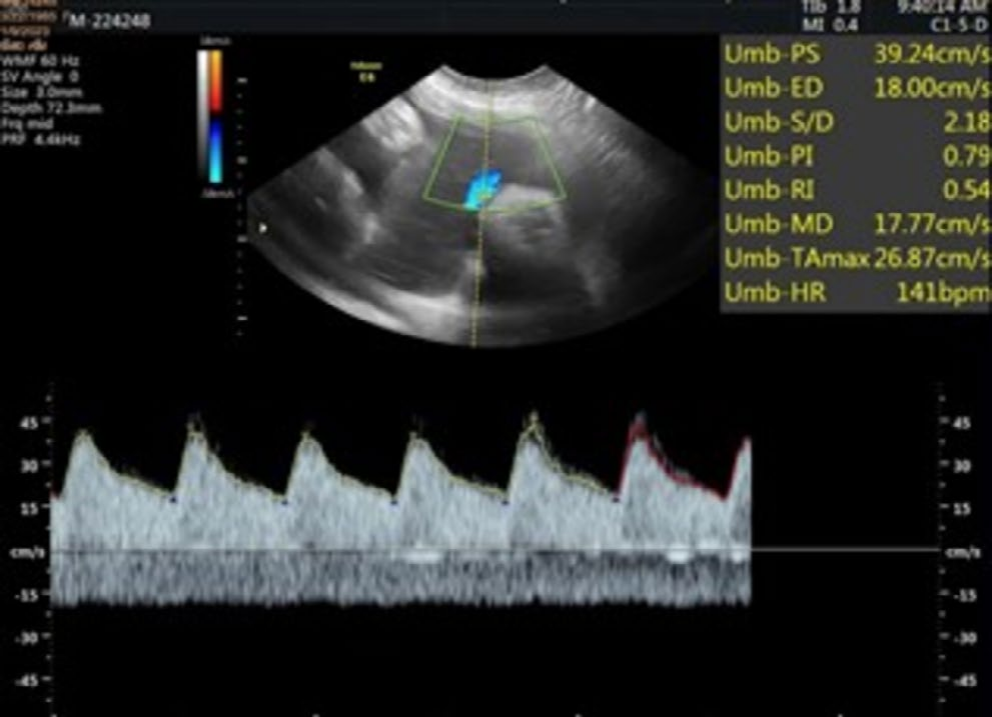
Fetal heart rate is 141 bpm, which appears regular with no gross cardiac structural abnormalities.

**FIGURE 2:**
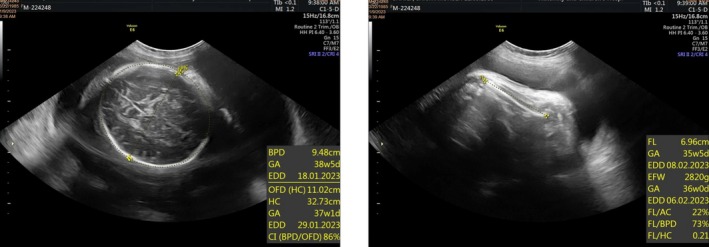
Fetal biometry showing growth parameters with normal ranges.

**FIGURE 3:**
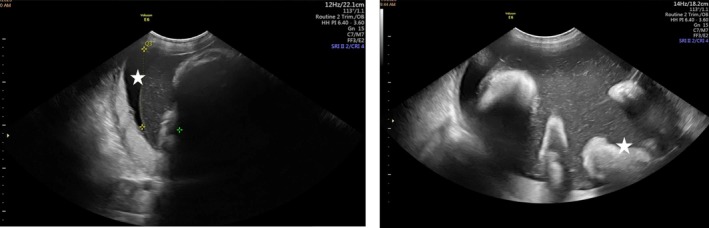
Reduced amniotic fluid volume with an AFP measurement of 38.8 cm, which is below the expected threshold for gestational age (left). Generalized hyper‐echogenicity of the skin suggesting thickened or parchment‐like skin (star), also breech presentation and partially imaged anterior placenta (right).

Subsequent follow‐up visits were uneventful, with the patient remaining asymptomatic and reporting preserved fetal movements. A repeat ultrasound at 28 weeks and 3 days (±2 weeks) demonstrated a viable fetus with a heart rate of 140 bpm (Figure 1) and an estimated fetal weight of 1200 g (±15%) (Figure 2). The placenta had migrated anteriorly to the fundus, with no evidence of retroplacental hemorrhage. Part of the membranes appeared separated at the lower uterine segment, with partial coverage of the internal cervical ostium. The amniotic fluid volume was reduced but still within the lower limit of normal for gestational age. Generalized hyperechogenicity of the fetal skin was noted, suggesting thickened or parchment‐like skin (Figure 3). Doppler evaluation revealed a resistive index of 0.67.

Maternal laboratory investigations included glycated hemoglobin (HbA1c), fasting blood glucose (FBG), and 2 h postprandial glucose (2HPP), all of which were within normal limits. Hemoglobin was slightly low at 11.2 g/dL, and virology screening was negative. At 36 weeks of gestation, the patient received dexamethasone for fetal lung maturity. She remained asymptomatic, with no labor pains, vaginal bleeding, or leakage of fluid.

**FIGURE 4:**
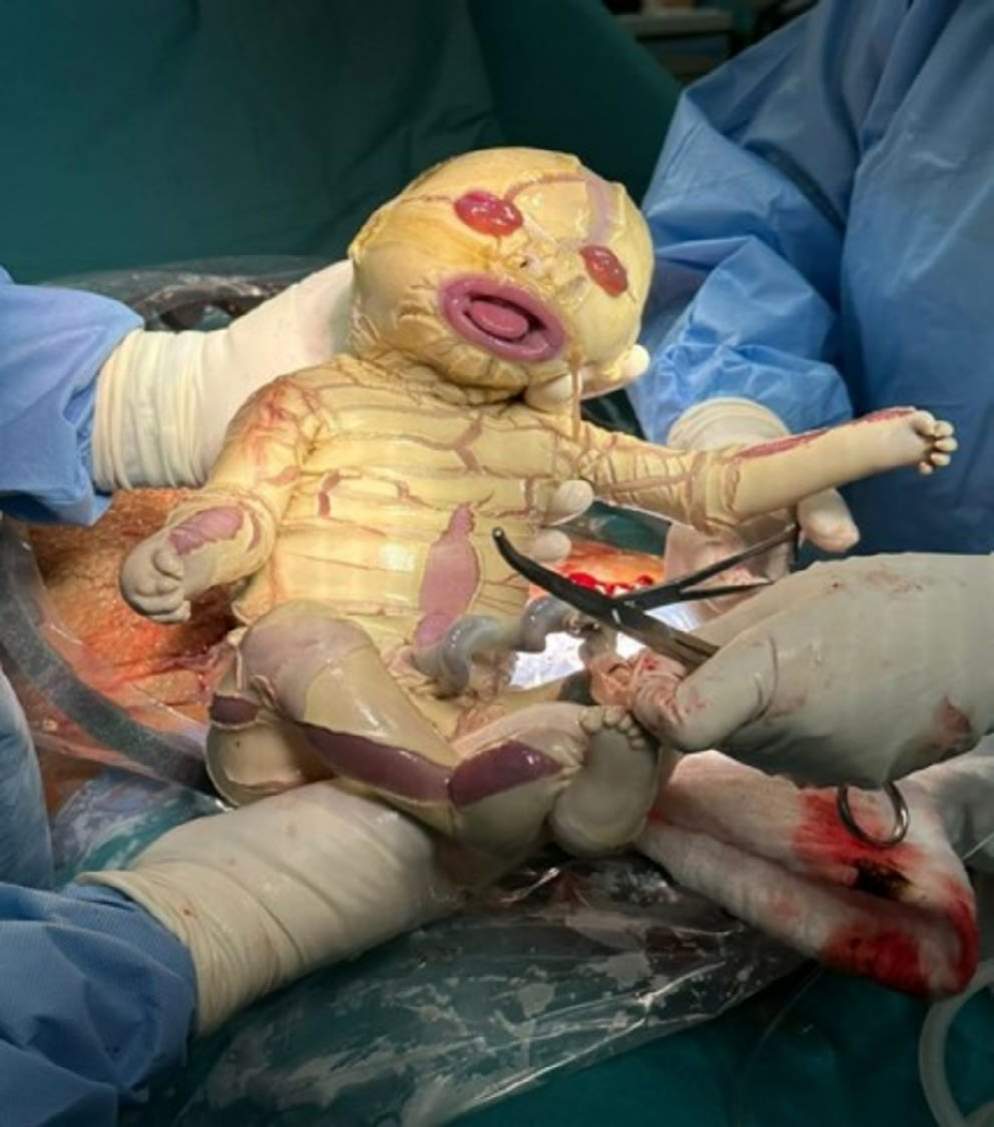
Image of the newborn showing cornification defects.

At 37 weeks, a lower‐segment cesarean section was performed for persistent breech presentation. A live male neonate weighing 2.5 kg was delivered with Apgar scores of 7 and 9 at 1 and 5 min, respectively. The newborn exhibited features of Harlequin ichthyosis, including thick, large, parchmentlike scales around the mouth and eyes, generalized cracked skin with deep fissures, severe ectropion, and eclabium producing a gaping “fishlike” appearance (Figure 4). The chest appeared rigid with restricted movement, and the limbs demonstrated pseudocontractures. No other external congenital anomalies were observed.

### 2.2 Investigations and Treatment

The neonate was admitted to the neonatal intensive care unit (NICU) for intensive supportive management. He was placed in a double‐walled incubator with a humidity of 60%–70% to minimize transepidermal water loss and prevent hypothermia. Temperature and fluid balance were continuously monitored.

Skin care included daily bathing with water and mild cleanser, followed by liberal application of emollients (Eucerin emollient cream and Aquaphor) every 3–4 h to soften the scales and prevent further fissuring. Sterile artificial tears were applied every 4 h to protect the exposed corneas. Nasogastric feeding was initiated early due to impaired oral feeding from eclabium.

Laboratory tests revealed leukocytosis (27.9 × 10^9^/L), elevated C‐reactive protein (91 mg/L; normal < 5 mg/L), thrombocytopenia (57 × 10^9^/L), and hyponatremia (121 mmol/L). Imaging studies, including echocardiography and abdominal and cranial ultrasonography, were unremarkable. Blood, umbilical, and skin swabs were collected for culture. Empiric intravenous ampicillin was administered while awaiting results.

Systemic retinoid therapy (oral acitretin) was considered but not initiated because of the infant's rapid deterioration and parental reluctance. Genetic testing for ABCA12 mutations was not performed due to the short survival period and the family's preference, which we acknowledge as a limitation.

### 2.3 Outcome and Follow‐Up

**FIGURE 5:**
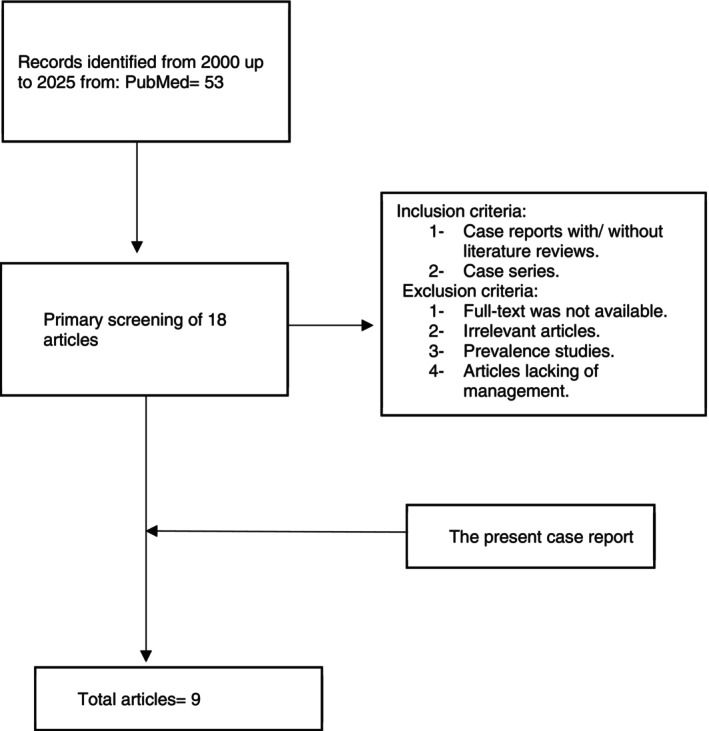
Flowchart of the literature review.

Despite intensive NICU care with optimal humidification, emollient therapy, infection control, and nutritional support, the infant developed progressive respiratory difficulty and electrolyte imbalance. He succumbed on the second day of life. The parents declined an autopsy (Figure 5).

## 3 Discussion

Harlequin ichthyosis (HI) is an extremely rare autosomal recessive congenital skin disorder, with a prevalence of approximately 1 in 300,000 live births. It carries a 25% recurrence risk in subsequent pregnancies [2, 6]. Over 90% of HI cases are caused by *ABCA12* mutations [7]. Defective ABCA12 function prevents lipid transport to the stratum corneum, resulting in a malformed epidermal barrier and massive hyperkeratosis. Histopathology shows a markedly thickened stratum corneum and absent lamellar granules [8]. The compromised skin barrier predisposes neonates to severe dehydration, electrolyte imbalances, and sepsis.

Clinically, HI neonates have a striking and pathognomonic appearance. They are often premature and encased in thick, shiny, armorlike hyperkeratotic plates, which soon crack into deep erythematous fissures [9]. These plates restrict limb and chest movement, contributing to respiratory compromise. Characteristic craniofacial features include severe ectropion, eclabium with a gaping “fishlike” mouth, a flattened nose, and hypoplastic or poorly formed ears [10]. Hands and feet are frequently encased in thick skin, producing pseudocontractures, and hair and nails are often abnormal [1]. Without prompt intervention, these infants typically succumb to respiratory failure or overwhelming sepsis. In a review of 45 clinical cases, Rajpopat et al. reported that 75% of deaths were attributed to sepsis and/or respiratory compromise [11].

Prenatal diagnosis of HI should be considered in families with a history of ichthyosis or neonatal deaths. Lategestation ultrasound may show characteristic findings such as a flattened face, fixed open mouth, dysplastic ears, and a “snowflake” pattern from desquamated skin particles in the amniotic fluid [12, 13]. Vijayakumari et al. reported a case where prenatal ultrasound at 28 weeks demonstrated intrauterine growth restriction, polyhydramnios, and facial anomalies, including an open mouth and absent nasal structures [14]. In our case, multiple antenatal ultrasounds were performed, revealing breech presentation and oligohydramnios; however, no craniofacial anomalies were detected due to fetal positioning. This highlights the diagnostic limitations of routine prenatal imaging in unsuspected cases of HI.

At birth, immediate and aggressive neonatal management is essential. The mainstay of therapy is supportive care, aiming to compensate for the absent skin barrier. Standard measures include placement in a high‐humidity incubator (50%–70%), careful temperature and fluid monitoring, infection prevention, and liberal use of bland emollients to reduce fissuring and transepidermal water loss [15]. Our patient received comprehensive NICU care, including humidification, emollient therapy, ocular lubrication, and early nasogastric feeding. Laboratory monitoring revealed leukocytosis, thrombocytopenia, hyponatremia, and elevated inflammatory markers, consistent with early sepsis risk [1, 16].

Systemic retinoid therapy, particularly oral acitretin, has improved the prognosis of HI in recent decades [10]. Retinoids promote desquamation, reduce hyperkeratosis, and can prevent ischemic complications of tight encasing plates [17]. Rajpopat et al. reported that 83% of infants treated with retinoids survived, compared with 24% in those who did not receive systemic therapy [11]. Topical retinoids, such as 0.1% tazarotene gel, may also provide local benefit in selected cases [18]. In our case, oral acitretin was considered but not initiated because of rapid clinical deterioration and parental reluctance.

We conducted a literature search in PubMed (2000–2025) and identified 12 recent case reports of congenital ichthyosis, including collodion baby (CB) and HI phenotypes. Among these, most neonates survived with early intensive care, and only one fatal case similar to ours was reported [19]. Table 1 summarizes the clinical features, management strategies, and outcomes of these cases. Our case underscores that although modern NICU care and retinoid therapy can improve survival, HI continues to carry a poor prognosis, particularly when diagnosis is delayed and systemic therapy is not initiated promptly.

**TABLE 1:** Summary of reported cases of congenital ichthyosis (2000–2025) including clinical features, management, and outcomes.

Study, year	Country	Sex	Highest APGAR	Survival outcome	Delivery	Treatment modality	Associated disorder	Consanguinity	Family history	Characterization of skin at birth	Diagnosis
Zdraveska, 2022 [20]	North Macedonia	Female	10	Survival at 8 years follow‐up	NVD	Placed in incubator with humidity set at 70%. Antibiotic treatment with ampicillin and amikacin	Claw hand deformity	No	Negative	Shiny, taut membrane with fissures and scaling	Self‐healing collodion baby
Quazi, 2023 [21]	India	Female	9	Discharged on improvement	NVD	Protection to the skin barrier, maintenance of fluid and electrolyte balance, and early initiation of retinoid therapy	No	No	Negative	Generalized parchment‐like membrane	CB
Karn, 2024 [22]	India	Female	—	Improvement at 2 weeks follow‐up	CS (at 36 weeks)	Releasing of taut membrane and amniotic membrane grafting	Bilateral ectropion	Yes	Positive	Thickened skin structure, ectropion and eclabium	CB
		Male	—	Improvement at 9 months	NVD (at 34 weeks)	Releasing of taut membrane and skin graft taken from mother		No	Negative	Tight, shiny, membrane‐like layer, similar to cellophane	CB
		Male	—	Improvement at 4 weeks	NVD (at 34 weeks)	Conservative management		Yes	Positive	Shiny, taut, cellophane‐like membrane	CB
		Male	—	NA	CS (at 36 weeks)	Conservative management		No	Positive	Milder form of lamellar ichthyosis	CB
		Male	—	NA	CS (at 36 weeks)	Conservative management		No	Positive	Milder form of lamellar ichthyosis	CB
Gulnerman,2022 [23]	Turkey	Male	10	Survival at 18 months	NVD (at 39 weeks + 6 days)	Oral acitretin 1 mg/kg/day. Parenteral nutrition via central venous catheterization	Ectropion and eclabium	No	Negative	Generalized thick translucent desquamation, including bilateral palmoplantar surfaces	CB with non‐bullous congenital ichthyosiform erythroderma
Bouab, 2023 [19]	Morocco	Female	8	Expired after 10 h	Premature at 32 weeks	Humidification in an incubator	No	No	Negative	Excessive scaling around the mouth having a typical fishlike appearance	CB
Munoz‐Aceituno, 2019 [24]	Spain	Male	—	Survived at 12 years	NVD	Topical keratolytic and oral retinoids	Loricrin keratoderma	No	Positive	Subtle shiny membrane over the child's entire body	CB
Besonhe, 2020 [25]	Belgium	Female	10	Survived at 4 months	—	Multidisciplinary team care	No	No	Positive	Bilateral ectropion, eclabium, stenosis of ear canals, and nasal vestibules	CB
Zhu, 2023 [26]	China	Female	9	Survival at 3 months follow‐up	NA	Sterile vaseline gauze dressings and maintained the air humidity	No	Yes	Negative	Taut, shiny, thick yellow crusts	Self‐improving collodion ichthyosis

Abbreviations: CB, collodion baby; CS, cesarean section; HI, Harlequin ichthyosis; NA, not available; NVD, normal vaginal delivery.

## 4 Conclusion

Harlequin ichthyosis is a rare and life‐threatening neonatal keratinization disorder that poses major diagnostic and management challenges. This case highlights the importance of thorough prenatal monitoring, early recognition of characteristic clinical features, and the prompt initiation of intensive supportive care, including humidity control, emollient therapy, and infection prevention. Despite these measures, the prognosis remains poor because of rapid fluid loss, respiratory compromise, and sepsis. Awareness of systemic retinoid therapy and the need for parental counseling are critical to improving outcomes and guiding the management of future pregnancies.

## Author Contributions

**Ahmed Alanzi:** conceptualization, data curation, investigation, supervision, writing – original draft, writing – review and editing. **Dawood Alatefi:** data curation, investigation, methodology, software, validation, writing – original draft, writing – review and editing. **Malik Alkabazi:** data curation, software, validation, writing – original draft, writing – review and editing. **Bano Alsaleh:** investigation, resources, validation. **Amir Fouad:** investigation, resources, visualization. **Islam Al Oraby:** investigation, resources, validation.

## Consent

Formal written informed consent for publication of this case report was obtained from the parents and it will be available upon request by the journal chief editor.

## Conflicts of Interest

The authors declare no conflicts of interest.

## Data Availability

The data used to support the findings of this study are included in the article.
